# Preliminary evidence for developing safe and efficient fecal microbiota transplantation as potential treatment for aged related cognitive impairments

**DOI:** 10.3389/fcimb.2023.1103189

**Published:** 2023-04-11

**Authors:** Xiaoxia Chen, Wanling Zhang, Zhijun Lin, Chunyan Zheng, Shufang Chen, Haihong Zhou, Zhou Liu

**Affiliations:** Department of Neurology, Guangdong Key Laboratory of Age-Related Cardiac and Cerebral Diseases, Institute of Neurology, Affiliated Hospital of Guangdong Medical University, Zhanjiang, Guangdong, China

**Keywords:** fecal microbiota transplantation, cognitive disorder, gut microbiota, metabolomics, safety

## Abstract

**Background:**

Recent studies have reported that gut microbiota is closely associated with cognitive fuction. Fecal microbiota transplantation (FMT) may be a potential treatment for cognitive impairment, but its efficacy in patients with cognitive impairment is unknown.

**Objectives:**

This study aimed to investigate the safety and efficacy of FMT for cognitive impairment treatment.

**Methods:**

Five patients aged 54–80 years (three women) were enrolled in this single-arm clinical trial from July 2021 to May 2022. The Montreal Cognitive Assessment-B (MoCA-B), Activities of Daily Living (ADL), and the cognitive section of the Alzheimer’s Disease Assessment Scale (ADAS-Cog) were assessed at days 0, 30, 60, 90, and 180. Additionally, stool and serum samples were obtained twice before FMT was administered and six months after the treatment. The structure of fecal microbiota was analyzed by 16S RNA gene sequencing. Serum samples were analyzed for metabolomics and lipopolysaccharide (LPS)-binding proteins by liquid chromatography-mass spectrometry and enzyme-linked immunosorbent assay, respectively. Safety was assessed based on adverse events, vital signs, and laboratory parameters during FMT and the follow-up period.

**Results:**

The MoCA, ADL, and ADAS-Cog scores of patients with mild cognitive impairment (patients C and E) after FMT were improved or maintained compared with those before transplantation. However, patients with severe cognitive impairment (patients A, B, and D) had no worsening of cognitive scores. Fecal microbiota analysis showed that FMT changed the structure of gut microbiota. The results of serum metabolomics analysis suggested that there were significant changes in the serum metabolomics of patients after FMT, with 7 up-regulated and 28 down-regulated metabolites. 3b,12a-dihydroxy-5a-cholanoic acid, 25-acetylvulgaroside, deoxycholic acid, 2(R)-hydroxydocosanoic acid, and P-anisic acid increased, while bilirubin and other metabolites decreased. KEFF pathway analysis indicated that the main metabolic pathways were bile secretion and choline metabolism in cancer. No adverse effects were reported throughout the study.

**Conclusions:**

In this pilot study, FMT could maintain and improve cognitive function in mild cognitive impairment by changing gut microbiota structure and affecting serum metabolomics. Fecal bacteria capsules were safe. However, further studies are needed to evaluate the safety and efficacy of fecal microbiota transplantation. ClinicalTrials.gov Identifier: CHiCTR2100043548.

## Introduction

Cognitive impairment refers to impairment of one or more cognitive areas, including memory, executive ability, language, attention, and visual space (McKhann et al., 2011), possibly leading to dementia. With the aging of the world population and the progress of medical science and technology, the world has seen a substantial increase in the diagnosis of cognitive disorders. Research has shown that about 35.6 million people worldwide suffered from dementia in 2010, and the number is expected to reach 115 million by 2050 (Prince et al., 2013).

Cognitive impairment is a complex multifactorial disease involving chronic neuroinflammation, dysfunction of the blood-brain barrier (BBB), and neurodegeneration (Raz et al., 2016). Furthermore, accumulating evidence has confirmed an association between dysbiotic gut microbiota and cognition disorder. Gut microbiota interacts with the central nervous system through the brain-gut axis. On the one hand, gut microbiota and its metabolites destroy the gut barrier function, allowing its harmful microorganisms and some neurotoxic metabolites to enter the central nervous system while activating the immune system and inflammatory cells, which affect the behavior and function of the brain (Zhang et al., 2017); (Mohajeri et al., 2018). On the contrary, the brain can also control gastrointestinal function (Jie et al., 2017). It has been pointed out that APP/PS1 transgenic mice treated with long-term high-dose antibiotic mixtures showed altered composition of gut microbiota and increased the neuroinflammatory state and therefore the disease itself (Angelucci et al., 2019).

Thus far, a number of studies have reported that probiotics and prebiotics reversed the cognitive behavioral deficits of AD mice and increased the diversity of gut microbiota and neurogenesis (Saulnier et al., 2013); (Jiang et al., 2017). Furthermore, fecal microbiota transplantation (FMT) provides a new therapeutic potential for patients with cognitive impairment. In animal experiments, AD mice (APP/PS1 double transgenic mice) demonstrated improved learning and memory ability after FMT. Fecal microbiota analysis has shown that the composition and structure of the gut microbiota between mice treated for FMT and normal control mice were more similar (Sun et al., 2019). Hazan et al. have reported a case of an AD patient treated with FMT (Hazan, 2020), in which the symptoms of cognitive impairment improved, and the Mini-Mental State Examination (MMSE) score increased from 20 to 26 (after 2 months) and 29 (after 6 months).

However, there are still few studies on cognitive impairment treatment with FMT, such as the choice of transplantation method, disease type, and disease course, which do not allow to recommend FMT for clinical application. More research is needed to answer these questions. Therefore, we conducted this study to understand the safety and efficacy of FMT in cognitive impairment treatment and provide a basis for further large sample studies of FMT in cognitive impairment treatment.

## Materials and methods

### Subject selection

Five patients diagnosed with cognitive impairment were recruited from the Affiliated Hospital of Guangdong Medical University. Inclusion criteria were (1) patients aged ≥ 50 years, (2) patients who met the diagnostic criteria for cognitive impairment, and (3) patients who signed written informed consent for this study. Exclusion criteria comprised (1) a history of short bowel syndrome and gastroesophageal reflux, (2) a history of recurrent aspiration, (3) low immunity, (4) disagreement to sign the informed consent, and (4) a history of taking probiotics and antibiotics within 1 month. No dietary changes or additional recommendations were made after FMT or during sample collection.

FMT capsules were produced by Xiamen Treatgut Biotechnology Co. Ltd. The donors were healthy college students around 20 years old, and screened through a series of questionnaires, clinical tests and metagenomic sequencing. Previous study have shown that Treatgut Biotechnology company (Xiamen,China) has recommended more stringent donor screening criteria for FMT in China (He et al., 2021). At least 100 g of fresh fecal samples was collected in a sterile plastic box within 1 hour after defecation. Fresh fecal sample (25%) should be mixed with normal saline (60%) and pharmaceutical grade glycerol (15%) in an automatic stirring separator. After mixing, each FMT capsule contained 1g of fecal mixture and was immediately frozen at - 80°C. Previously reported that combining meta-analysis and analytic hierarchy process, the imbalance characteristics of patients in microbial diversity, overall microbial structure, bacterial groups and metabolic pathways were identified, and a donor-recipient matching model based on these microbial characteristics was constructed to accurately guide donor selection in the process of microbial transplantation (Zhang et al., 2022).

### Study design and intervention

This was an open-label, single-arm clinical trial that received institutional approval from the Ethics Review Board of the Affiliated Hospital of Guangdong Medical University (PJ2020-110) and was conducted in accordance with the Helsinki statement. All patients provided informed consent. If the patient was eligible, they took fecal microbiota capsules three times. Before use, the device was incubated at 37°C for 10 min and then placed at room temperature for 10 min. The patients fasted 6 hours before and 1 hour after FMT. FMT was taken within 3 hours, with a total of 40 pills per intake. Patients were required to return to the hospital for FMT every other week. Indicators of cognitive ability, including Activities of Daily Living (ADL), the Montreal Cognitive Assessment-B (MoCA-B), and the cognitive section of the Alzheimer’s Disease Assessment Scale (ADAS-Cog) were assessed at days 0, 30, 60, 90, and 180. Fecal and serum samples were also collected at days 0 and 180.

### Cognitive assessment

All people who assessed patients’ cognitive function were professionally trained. The baseline and follow-up cognitive scales were assessed by the same professional physician. As cognitive function assessment methods, ADAS-Cog, ADL, and MoCA-B were used. ADAS-Cog scale included 9 functional sub-tests (orientation, comprehension, memory, and execution of orders) and 2 memory sub-tests (words recall and recognition). ADL scale included 20 items, such as eating, dressing, bathing, cooking, going to and from the toilet, walking around the room, urine and defecation control, washing, moving around the neighborhood, taking medicine, cooking by yourself, doing housework, shopping, managing money, and using mobile phones. MoCA scale, the Chinese version of the Basic Scale (MOCA-B), covered executive function, fluency, orientation, memory, abstraction, delayed recall, visual perception, naming, and attention.

### ELISA assay

Human lipopolysaccharide (LPS)-binding protein (LBP) was determined in serum samples using an ELISA kit (Hycult^®^Biotech) according to the instructions provided by the manufacturer. Absorbance was read at 450 nm by a microplate reader and then calculated as concentration based on a standard curve.

### 16S RNA gene sequencing

16S DNA sequencing was completed by Xiamen Treatgut Biotechnology Co. Ltd. DNA was extracted from approximately 0.25 g of fecal samples using the QIAamp Fast DNA Stool Mini Kit (Qiagen, CA, USA) according to the manufacturer’s instructions. The concentrations and purity of the isolated DNAs were assessed using spectrophotometry (Multiskan™ GO, Thermo Fisher Scientific, USA). The DNA extracts also were evaluated for quality by agarose (1.5%) gel electrophoresis in 1× Tris-Acetate-EDTA buffer. Samples were stored at −20˚C before being used as templates for next-generation sequencing library preparation.

The V4 region of the 16S rRNA gene was amplified using the specific primers (515F [5’-GTGCCAGCMGCCGCGGTAA-3’] and 806R [5’-GGACTACNVGGGTWTCTAAT-3’]) with the barcode. The same volume of 1× loading buffer (contained SYB green) was mixed with polymerase chain reaction (PCR) products, followed by 2% agarose gel electrophoresis. The samples with the bright main strip between 400–450 bp were chosen for further experiments. PCR products were mixed in equidense ratios. Then, a mixture of PCR products was purified with GeneJET Gel Extraction Kit (Thermo Scientific). Sequencing libraries were generated using TruSeq^®^ DNA PCR-Free Sample Preparation Kit (Illumina) following the manufacturer’s recommendations, and index codes were added. The library quality was assessed on the Qubit@ 2.0 Fluorometer (Thermo Scientific) and Agilent Bioanalyzer 2100 system. Lastly, the library was sequenced on an Illumina MiniSeq, and 150 bp paired-end reads were generated.

### Analysis of serum metabolome by liquid chromatography-mass spectrometry (LC-MS)

One hundred µl liquid samples were accurately weighed, and the metabolites were extracted using a 400-µL methanol:water (4:1, vol/vol) solution. The mixture was allowed to settle at −20°C and treated by high throughput tissue crusher Wonbio-96c (Shanghai Wanbo Biotechnology Co., Ltd.) at 50 Hz for 6 min, followed by vortex for 30 s and ultrasound at 40 kHz for 30 min at 5°C. The samples were placed at −20°C for 30 min to precipitate proteins. After centrifugation at 13000g at 4°C for 15 min, the supernatant was carefully transferred to sample vials for LC-MS/MS analysis.

After LC-MS/MS analyses, the raw data were imported into the Progenesis QI 2.3 (Nonlinear Dynamics, Waters, USA) for peak detection and alignment. Metabolic features detecting at least 80% in any set of samples were retained. After filtering, each metabolic feature was normalized by sum. The internal standard was used for data quality control (QC) (reproducibility). Metabolic features with a relative standard deviation (RSD) of QC of > 30% were discarded. Mass spectra of these metabolic features were identified by using the accurate mass, MS/MS fragments spectra, and isotope ratio difference by searching in reliable biochemical databases, such as the Human Metabolome Database (HMDB) (http://www.hmdb.ca/) and Metlin database (https://metlin.scripps.edu/).

The preprocessed data were analyzed on the Meggie Biocloud platform (https://cloud.majorbio.com). Ropls (version 1.6.2) R package was used for principal component analysis (PCA) and orthogonal partial least squares discriminate analysis (OPLS-DA), and the stability of the model was evaluated by cross-validation with seven cycles. Additionally, Student’s t test and differential multiple analysis were performed. The selection of significantly different metabolites was determined based on the variable weight value (VIP) obtained by the OPLS-DA model and the *P* value of student’s t test. VIP of ≥ 1 and *P* of > 0.05 were considered significantly different metabolites. Differential metabolites were annotated through the KEGG database (https://www.kegg.jp/kegg/pathway.html) for metabolic pathways.

### Safety assessments

For each patient, safety was assessed based on adverse events (including death or drop-out), vital signs, and laboratory parameters including blood routine examination and blood pathogens such as toxoplasma gondii, rubella virus, cytomegalovirus, herpes simplex virus and syphilis during FMT and the follow-up period.

### Statistical analysis

SPSS 20.0 and Prism 8.0 were used for data analysis. Measurement data were expressed as mean ± standard deviation ( $\bar{x}\pm s$ ). Kruskal-Wallis test, Wilcoxon test, and t test were used for comparison between groups. The Chi-square test was used to compare the differences between groups. *P* of <0.05 was considered statistically significant.

## Results

### Baseline information

Five patients with cognitive impairment (AD, n = 2; mild cognitive impairment, n = 2; frontotemporal dementia, n = 1) underwent this pilot study (**Table 1**).

**Table 1:** Basic information of patients with cognitive impairment.

	A	B	C	D	E
Age, y	74	68	77	54	80
Sex	Male	Female	Female	Female	Male
Education	Middle school	Junior college	High School	Primary school	Junior college
BMI, kg/cm^2^	20	23.1	20	22.2	22.5
Illness course, y	3	5	2	3	2
Complicating disease	None	Diabetes	Anxiety disorder	None	Chronic gastritis
MMSE	11	6	27	8	27
MoCA	4	3	21	3	17
ADL	46	41	22	24	20
ADAS-Cog	43	62	14	53	10
Diagnosis	Alzheimer disease	Frontotemporal dementia	Mild cognitive impairment	Alzheimer disease	Mild cognitive impairment

### Changes in cognition and daily living ability after FMT

**Figure 1:**
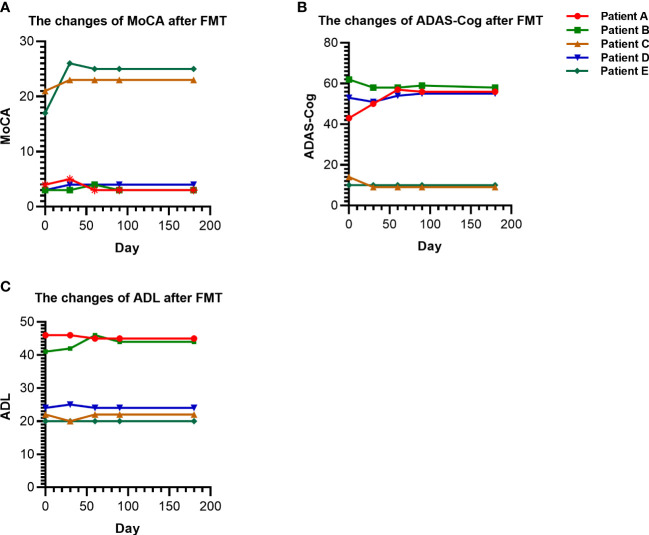
Changes in cognition and ability of daily living scores before and after FMT. **(A)** The changes of MoCA in patients before and after FMT. **(B)** The changes of ADAS-Cog in patients before and after FMT. **(C)** The changes of ADL in patients before and after FMT. FMT, Fecal microbiota transplantation.

As shown in **Figure 1**, the MoCA scores of patients with mild cognitive impairment (patients C and E) after FMT did not significantly change or slightly increased compared with those before transplantation, while the ADL and ADAS-Cog scores decreased. The cognitive scores of AD (patients A and D) were basically maintained. However, the cognitive score of frontotemporal dementia (patient B) remained stable without aggravation after FMT.

### Changes of LPS binding proteins after FMT

As shown in **Table 2**, the LBP of patients was lower than that before FMT, but the difference was not statistically significant (*P* > 0.05).

**Table 2:** Changes of LBP before and after fecal bacterial transplantation.

	Before FMT	After FMT	*P*
LBP (ng/ml)	46.30 ± 0.95	45.77 ± 1.42	0.50

### FMT regulated the structure of gut microbiota

**Figure 2:**
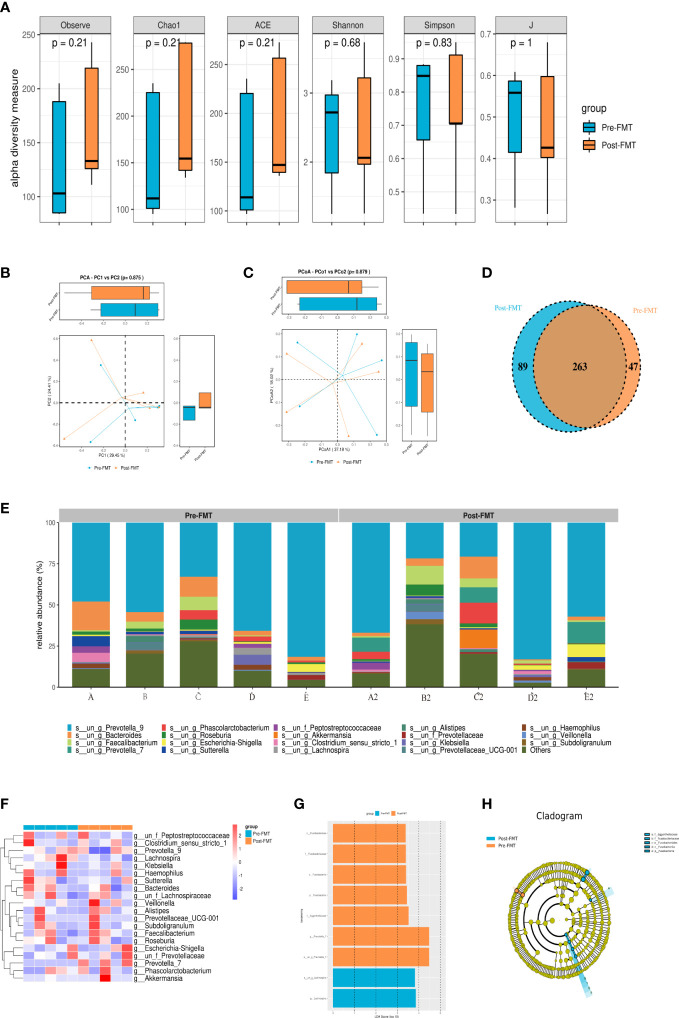
Results of 16S rRNA high-throughput sequencing analysis of gut microbiota before and after FMT. **(A)** Alpha diversity analysis of gut microbiota in patients before and after FMT. **(B)** PCA diversity analysis of gut microbiota in patients before and after FMT. **(C)** PCoA analysis of gut microbiota in patients before and after FMT. **(D)** OTU Venn diagram of gut microbiota in patients before and after FMT. **(E)** Community composition analysis of gut microbiota in patients before and after FMT. **(F)** Heatmap of gut microbiota in patients before and after FMT. **(G, H)** LEfSe analysis of gut microbiota in patients before and after FMT.

Alpha diversity could reflect richness (ACE, Chao1, and Observed species), diversity (Shannon and Simpson), and evenness (J) of microbial communities within the sample. **Figure 2A** reveals that the alpha diversity of gut microbiota in patients after FMT increased compared with that before transplantation, but *P* was > 0.05. No significant difference was observed in the beta diversity which represented by PCA and principal-coordinate analysis (PCoA) (P > 0.05; **Figures 2B, C**). Veen diagram showed that there were 263 species of the same gut microbiota before and after FMT in patients with cognitive impairment, while 47 species were unique before FMT and 89 species were unique after FMT (**Figure 2D**).

As shown in **Figure 2E**, *g_Prevotella_9* and *g_Bacteroides* accounted for a higher proportion of fecal microbiota composition before and after transplantation in each patient. Interestingly, the composition of fecal microbiota was significantly different among patients. *g_Prevotella_9* in the gut microbiota of patients A and D after capsule administration was lower than that before transplantation, while *g_Prevotella_9* in the gut microbiota of patients B, C and E had higher abundance than that before FMT. Additionally, the percentage of *g_Bacteroides* in the gut microbiota of patients A, B and D after FMT was lower than that before transplantation, while the opposite result was found in patients C and E.

After standardization according to the operational taxonomic unit (OTU), the top 30 species with the largest number were selected, and then heat maps were created. The correlation method was selected by default for the clustering distance of species in the heat map. Each color block in the heatmap represented the abundance of a taxonomic level for a sample, and different colors indicated different proportions of species abundance. **Figure 2F** shows that the trend of gut microbiota composition structure was consistent with the above-described results.

Linear discriminant analysis Effect Size (LEfSe) was used to compare multiple groups, and subgroup comparison was performed within the group comparison. Linear discriminant analysis (LDA) was used to reduce the data and evaluate the influence of significantly different species (LDA score) to find the species with significantly different abundance between groups. After FMT, the patients were rich in *o_Fusobacteriales, f_Fusobacteriaceae*, *c_Fusobacteriia*, *p_Fusobacteria*, *f_Eggerthellaceae*, and *g_Prevotella_7*. However, *s_un_g_Lachnospira* and *g_Lachnospira* were decreased (**Figures 2G, H**).

### FMT regulated serum metabolome.

A total of 512 cation peaks, 271 anion peaks, 386 positive metabolites, and 222 negative metabolites were detected in serum before and after FMT by LC/MS mass spectrometry.

**Figure 3:**
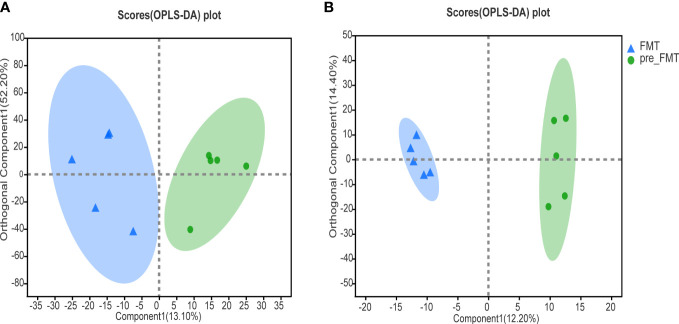
Heatmap of serum metabolomics correlation before and after FMT. **(A)** OPLS-DA analysis of cation profiles. **(B)** OPLS-DA analysis of anion table.

OPLS-DA could better identify the differences between groups and improve the effectiveness and analytical ability of the model. As shown in **Figure 3**, the samples of patients before and after FMT were separated, suggesting that the metabolomics of patients before and after FMT had obvious changes.

**Figure 4:**
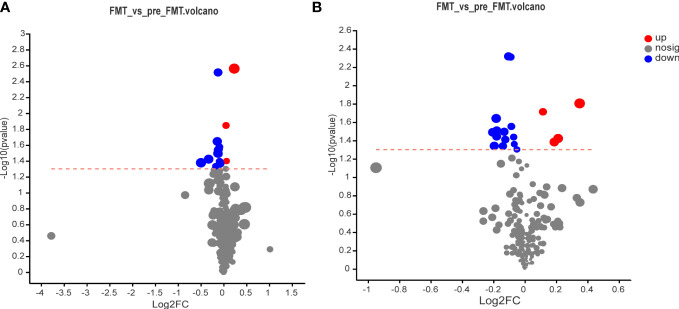
Volcano map of serum metabolomics before and after FMT. **(A)** Volcano map of cation surface. **(B)** Volcano diagram of anion table.

The volcano map illustrated the up-and down-regulated serum metabolites before and after FMT, with each dot representing a specific metabolite and the size of the dot indicating the VIP value. After FMT, 7 metabolites were up-regulated and 28 metabolites were down-regulated (**Figure 4** **).**

**Figure 5:**
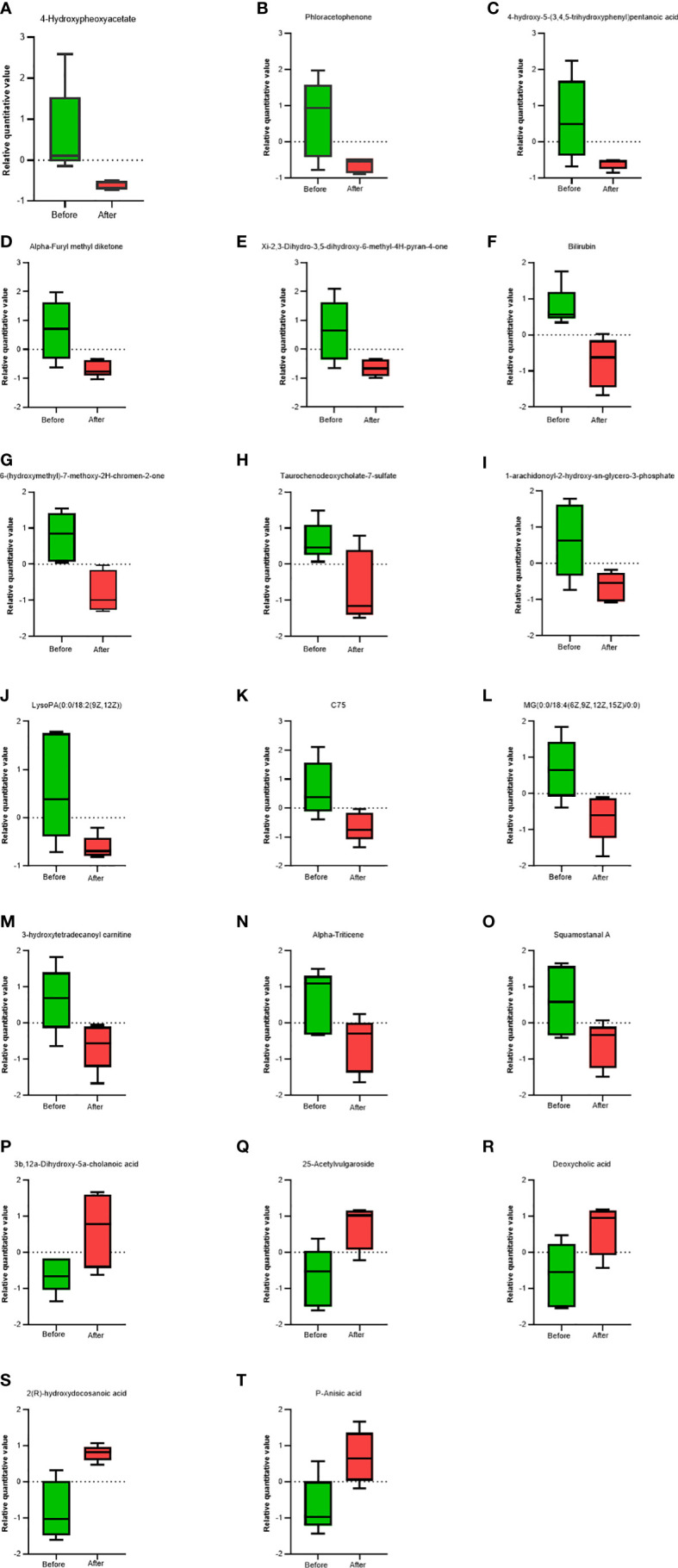
Comparison of serum metabolites before and after FMT. Box charts showed statistical difference in the 4-Hydroxypheoxyacetate **(A)**, Phloracetophenone **(B)**, 4-hydroxy-5-(3,4,5-trihydroxyphenyl)pentanoic acid **(C)**, Alpha-Furyl methyl diketone **(D)**, Xi-2,3-Dihydro-3, 5-dihydroxy-6-methyl-4H-pyran-4-one **(E)**, Bilirubin **(F)**, 6-(hydroxymethyl)-7-methoxy-2H-chromen-2-one **(G)**, Taurochenodeoxycholate-7-sulfate **(H)**, 1-arachidonoyl-2-hydroxy-sn-glycero-3-phosphate **(I)**, LysoPA(0.0/18:2(9Z, 12Z)) **(J)**, C75 **(K)**, MG(0:0/18:4(6Z,9Z,12Z,15Z)/0:0) **(L)**, 3-hydroxytetradecanoyl carnitine **(M)**, Alpha-Triticene **(N)**, Squamostanal A **(O)**, 3b,12a-Dihydroxy-5a-cholanoic acid **(P)**, 25-Acetylvulgaroside **(Q)**, Deoxycholic acid **(R)**, 2(R)-hydroxydocosanoic acid **(S)**, P-Anisic acid **(T)**.

**Figure 6:**
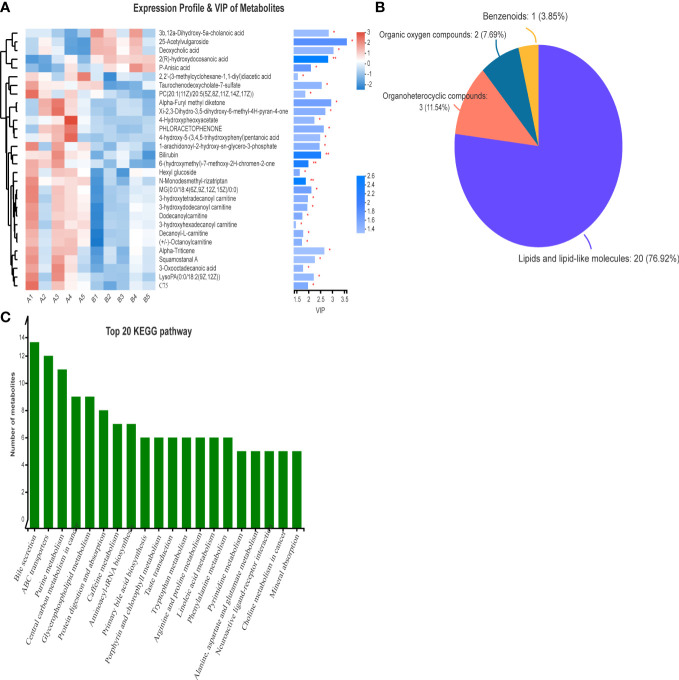
VIP analysis and HMDB compound classification of serum metabolomics before and after FMT. **(A)** VIP analysis of serum metabolomics before and after FMT. **(B)** Compound classification map of serum metabolomics before and after FMT. **(C)** KEGG pathway analysis of serum metabolomics in patients before and after FMT.

VIP analysis showed the expression patterns of different metabolites in each sample, *P* values of metabolites in unidimensional statistics, and VIP values in multivariate statistical analysis to directly observe the importance of different metabolites and the trend of their expression. As shown in **Figure 5**, **Figure 6A**, the top 20 metabolites with VIP value were selected for analysis. The results revealed that bilirubin, 4-Hydroxypheoxyacetate, Phloracetophenone, 4-hydroxy-5-(3,4,5-trihydroxyphenyl)pentanoic acid, Alpha-Furyl methyl diketone, Xi-2,3-Dihydro-3,5-dihydroxy-6-methyl-4H-pyran-4-one, 6-(hydroxymethyl)-7-methoxy-2H-chromen-2-one, Alpha-Triticene, Squamostanal A and other metabolites decreased after FMT. In contrast, 3b,12a-Dihydroxy-5a-cholanoic acid, 25-Acetylvulgaroside, Deoxycholic acid, 2(R)-hydroxydocosanoic acid and P-Anisic acid increased compared with those before transplantation.

The differences in metabolites before and after FMT were classified into compounds. **Figure 6B** shows that the main differentially expressed metabolites after FMT were lipids and lipid molecules (76.92%), organic heterocyclic compounds (11.54%), and organic oxygen compounds (7.69%). At the same time, the pathways of differential metabolites mainly came from bile secretion, choline metabolism, reverse nerve signaling, linoleic acid metabolism, linolenic acid metabolism, and glycerophospholipid metabolism (**Figure 6C**).

### Safety of FMT

During the overall study period, the five patients did not experience adverse events, including abdominal pain, diarrhea, nausea, fever, infection, headache, cardiac event, and aggravation of illness. There were no deaths or serious adverse events (**Table 3**).

**Table 3:** Adverse events during overall follow-up time.

Adverse events	Patients, No. (%)
Any adverse events	0
Serious adverse events	0
Abdominal pain	0
Diarrhea	0
Fever	0
Infection	0
Headache	0
Nausea	0
Cardiac event	0
Aggravation of illness	0
Death	0

## Discussion

Recent studies have shown that gut microbiota plays an important role in degenerative diseases. However, no clinical trials of FMT for cognitive impairment have been reported. In this study, we found that FMT treatment could maintain stability or even alleviate cognitive impairment and change the structure of gut microbiota, metabolome, and metabolic pathways.

Cognitive impairment is the main clinical manifestation of dementia (Owen et al., 2009); (Steel, 2010). We found that the cognitive scores of patients with mild cognitive impairment after FMT were improved or maintained compared with those before transplantation. In fact, an increasing number of animal experiments and case reports have also verified that FMT can improve cognitive deficits (Yu et al., 2019); (Sun et al., 2019). Different from our results, the gastrointestinal condition, cognitive condition, daily living ability, and emotion of AD patients with recurrent *Clostridium difficile* infection have been significantly improved after FMT treatment (Gotoh et al., 2021). This might be related to the severity of cognitive impairment, mode of FMT administration, storage time of fecal suspension, and the use of antibiotics. Additionally, we also found that the improvement of cognitive deficits in patients with mild cognitive impairment was more significant, which might suggest that it is difficult for FMT to reverse the course of severe cognitive impairment. On the other hand, inflammatory responses, especially gut inflammation and neuroinflammation, are considered key factors in dementia pathogenesis. A double-blind, randomized trial has found that higher LBP translocated from the gut to the blood caused persistent attention deficits and cognitive impairment (Madison et al., 2020). Another study has also revealed that fecal transplants of mice from patients with cognitive impairment after stroke showed worse cognitive function, higher levels of LBP and gut toll-like receptor 4, and more severe gut disruption (Wang et al., 2022). We found that LBP was lower after FMT than before transplantation, but the difference was not statistically significant. This might be caused by the small sample size, long sample storage, and short follow-up observation time. In this study, these results suggested that FMT might maintain cognitive impairment symptoms and improve “leaky gut” responses.

Previous studies have demonstrated that gut microbiota influences CNS function and metabolism through the gut-brain axis. Zhan et al. have suggested that different compositions of the gut microbiota might contribute to cognitive differences between senescence-accelerated mouse prone 8 (SAMP8) and senescence-accelerated mouse resistant 1 (SAMR1) mice. They have also found that pseudo-germ-free mice that received fecal bacterial transplantation from SAMR1 mice demonstrated improved cognitive behaviors, such as spatial learning and memory. At the same time, the intestinal diversity was increased, and the level of beneficial bacteria, such as *Paraclactobacillus* was increased (Zhan et al., 2018). Other studies have also suggested that FMT could directly reconstruct gut microbiota (Fillon, 2021); (Mocanu et al., 2021). The results of our experiment supported this view, in which patients who took fecal microbiota capsules had an improved structure of gut microbiota. Furthermore, community composition analysis showed that the proportion of gut microbiota community composition in patients before and after transplantation was different, among which *Prevotella* accounted for the highest proportion. After FMT, *c_Fusobacteriia*, *g_Provotella_7*, *s_un_g_Provotella_7*, *f_Eggerthellaceae*, and other bacteria increased, while *G_Lachnospira* and *s_un_g_Lachnospira* decreased. *Prevotella* could produce anti-inflammatory metabolites propionic acid and butyric acid, reducing proinflammatory response (Esquivel-Elizondo et al., 2017). Kang et al. have believed that the levels of *Prevotella* and *Bifidobacteria* increased after FMT in patients with Autism Spectrum Disorders (ASD), and the autism-related symptoms were significantly improved. Notably, the abundance of *Bifidobacteria* and *Prevotella* was still significantly increased after the end of the follow-up (Kang et al., 2019). Interestingly, *Fusobacteria*, Gram-negative bacteria, were associated with bradykinesia and social difficulties and could increase the incidence of gastrointestinal injury and diseases (Yang et al., 2021). The abundance of *Eggerthellaceae* had a strong relationship with lipid metabolism and bile acids (Wen et al., 2020); (Zhao et al., 2014). A recent study has proposed that *Eggerthellaceae, Rodentibacter, Bacteroides, Ruminococcaceae_UCG_014, Faecalibaculum*, and *Muribaculaceae* were increased after surgery in APP/PS1 mice with cognitive impairment (Han et al., 2020; Guo et al., 2021). However, we found different results. In another study, the number of *Lachnospira*, *Bacteroides*, and *Ruminiclostridium_9* in patients with AD was significantly lower than in healthy controls, while the number of *Prevotella* in AD patients was higher than in healthy controls (Guo et al., 2021).

According to our study, the differential metabolites mainly concentrated in bile secretion, choline metabolism, linolenic acid metabolism, phospholipid metabolism, and linoleic acid metabolism. Compounds secreted or affected by the gut microbiota lead to neurodegeneration through the blood-brain barrier (BBB). Recent evidence has shown that bile acids have protective and anti-inflammatory effects on the brain. Primary bile acids are decreased in AD and MCI patients, while secondary bile acids, such as taurodeoxycholic acid and deoxycholic acid produced by gut microbiota, are increased compared with normal controls (Olazarán et al., 2015). Liu et al. have believed that secondary bile acids could improve cognitive deficits and maintain homeostasis of glucose and lipid metabolism (Liu et al., 2020). In our study, the metabolites of deoxycholic acid and some cholic acids, such as 3b,12a-dihydroxy-5a-cholanoic acid, were increased after FMT. Previous studies have pointed out that gut microbiota (*Bacteroides*, *Fusobacteria*, and *Firmicutes*) regulate bile metabolism, glucose metabolism, amino acid metabolism, and phospholipid metabolism by affecting the farnesoid X receptor (FXRα) and the cell membrane receptor G-protein coupled receptor 5 (TGR5) (Liu et al., 2020). Additionally, free bilirubin increases the risk of AD, which can aggravate neurotoxicity and induce neuronal damage, tau protein hyperphosphorylation, and deposition of Aβ by activating glycogen synthase kinase-3β (GSK-3β), c-Jun amino terminal kinase (JNK), and cyclin-dependent kinase 5 (CDK5) (Staley et al., 2017); (Kimpara et al., 2000). In this study, the content of serum bilirubin significantly decreased after FMT. Drzazga et al. have proposed that anisic acid combined with lysophosphatidyl choline could promote insulin secretion by targeting G-coupled receptors (Drzazga et al., 2020). Diabetes increases the risk of cognitive impairment. Hence, anisic acid might improve cognitive impairment by reducing the risk of diabetes. Moreover, higher concentrations of plasma choline are converted to trimethylamine-N-oxide (TMAO) by the gut microbiota, which increases the risk of cardiovascular diseases and cognitive deficits (Lee et al., 2021). Linoleic acid, linolenic acid, and phospholipid metabolism belong to lipid metabolism, and previous studies have suggested that disorders of lipid metabolic pathways were related to AD pathogenesis (Bowers et al., 2020). These metabolic pathways have anti-inflammatory effects and, thus, reduce Aβ aggregation. LysoPC, a product of phospholipid metabolism, can lead to the deposition of Aβ in the brain and increases neurotoxicity (Zhang et al., 2020). This suggested that FMT might affect cognition by producing or affecting metabolites. However, the mechanism by which microorganisms affect cognitive impairment through metabolic pathways has not been clarified and still needs to be further studied.

This experiment had certain limitations. First, the sample size was small. Second, there was a lack of a control group based on the results obtained through self-control before and after the treatment. Third, both gut microbiota and metabolites might be affected by region and dietary habits, limiting the generalizability of our findings.

## Conclusion

In conclusion, FMT altered the structure of gut microbiota. On the other hand, FMT affected the pathogenesis of dementia by changing metabolites or metabolic pathways. Fecal bacteria capsules were safe. Further validation is needed in randomized, double-blind, controlled clinical trials.

## Data availability statement

The metabolomics data presented in the study are deposited in the Figshare repository, https://doi.org/10.6084/m9.figshare.21755357.v1. The 16S RNA gene sequencing data are deposited in the NCBI Sequence Read Archive under accession number PRJNA910861.

## Ethics statement

The studies involving human participants were reviewed and approved by the Affiliated Hospital of Affiliated Hospital of Guangdong Medical University. The patients/participants provided their written informed consent to participate in this study.Written informed consent was obtained from the individual(s) for the publication of any potentially identifiable images or data included in this article.

## Author contributions

ZLiu, HZ and XC conceived and designed the clinical trial. XC, WZ, ZJL, CZ and SC made substantial contributions to sampling, processing sample, assessing cognitive scales, and managing participants. ZLiu and XC analyzed or interpreted the data, drafted and reviewed the manuscript. All authors contributed to the article and approved the submitted version.
